# AI models for automated segmentation of engineered polycystic kidney tubules

**DOI:** 10.1038/s41598-024-52677-1

**Published:** 2024-02-03

**Authors:** Simone Monaco, Nicole Bussola, Sara Buttò, Diego Sona, Flavio Giobergia, Giuseppe Jurman, Christodoulos Xinaris, Daniele Apiletti

**Affiliations:** 1 DAUIN, Politecnico di Torino, 10129 Turin, Italy; 2 Fondazione Bruno Kessler, 38123 Trento, Italy; 3 CIBIO, Università degli Studi di Trento, 38123 Trento, Italy; 4 Istituto di Ricerche Farmacologiche Mario Negri - IRCCS, 24126, Bergamo, Italy

**Keywords:** Data mining, Machine learning

## Abstract

Autosomal dominant polycystic kidney disease (ADPKD) is a monogenic, rare disease, characterized by the formation of multiple cysts that grow out of the renal tubules. Despite intensive attempts to develop new drugs or repurpose existing ones, there is currently no definitive cure for ADPKD. This is primarily due to the complex and variable pathogenesis of the disease and the lack of models that can faithfully reproduce the human phenotype. Therefore, the development of models that allow automated detection of cysts’ growth directly on human kidney tissue is a crucial step in the search for efficient therapeutic solutions. Artificial Intelligence methods, and deep learning algorithms in particular, can provide powerful and effective solutions to such tasks, and indeed various architectures have been proposed in the literature in recent years. Here, we comparatively review state-of-the-art deep learning segmentation models, using as a testbed a set of sequential RGB immunofluorescence images from 4 in vitro experiments with 32 engineered polycystic kidney tubules. To gain a deeper understanding of the detection process, we implemented both pixel-wise and cyst-wise performance metrics to evaluate the algorithms. Overall, two models stand out as the best performing, namely UNet++ and UACANet: the latter uses a self-attention mechanism introducing some explainability aspects that can be further exploited in future developments, thus making it the most promising algorithm to build upon towards a more refined cyst-detection platform. UACANet model achieves a cyst-wise Intersection over Union of 0.83, 0.91 for Recall, and 0.92 for Precision when applied to detect large-size cysts. On all-size cysts, UACANet averages at 0.624 pixel-wise Intersection over Union. The code to reproduce all results is freely available in a public GitHub repository.

## Introduction

The recent onset of a continuous symbiosis between biotechnology and artificial intelligence (AI) has proven to be particularly effective in areas of life sciences where classical approaches are not an option. Drug discovery is one of such activities, especially for diseases whose treatment options are dramatically limited, due to the intrinsic nature of the pathology as well as the lack of models that can faithfully reproduce the human phenotype. This is indeed the case here, where a novel drug testing strategy is introduced, combining an *in vitro* experimental setup with deep learning modeling into a pipeline aimed at accelerating the testing of novel compounds. The target condition is the Autosomal Dominant Polycystic Kidney Disease (ADPKD), the most common inherited monogenic kidney disorder that affects $1/500 - 1/2500$ individuals^1,2^ worldwide. ADPKD distinctive phenotype is the formation and the progressive growth of multiple cysts that gradually replace the kidney parenchyma, thus leading to an impairment of kidney structure and function and, eventually, to end-stage kidney disease^3^. In particular, cytogenesis involves a large number of diverse signaling cascades and pathways, such as PC1/2 (polycystin-1 and 2) signaling, cilia-related cascades, and growth factors-related signaling^4^.

Apart from the conventional anti-hypertensive strategies^5^, there are currently two drugs that have recently been repurposed and used sometimes to reduce the growth rate of cysts in ADPKD: Tolvaptan and Octreotide-LAR^6^. However, these drugs are only available to patients at high risk of end-stage kidney disease (ESKD), while an important number of ADPKD patients progress to ESKD despite the treatments. There is currently no definitive cure for ADPKD. This is mainly due to the high phenotypical and genotypical variability between patients, the complex pathobiology of the disease, and the lack of models that can faithfully mimic the human phenotype.

As such, there is an urgent need for developing patient-specific models that can replicate key aspects of polycystic kidneys and be used for drug testing studies. Using 3D printing technologies and patients’ cells, we have developed a platform that has enabled us to study the pathogenesis of the disease directly in human tissues and to identify novel therapeutic targets^7,8^. In essence, this system based on engineered kidney tubules displays significant advantages over previous 2D models: (i) it provides patient-specific 3D tissues with polarized epithelium and lumen that can be quantifiably and quantitatively used to study drug toxicity on tissue anatomy, integrity, and anticystogenic efficacy; (ii) it allows evaluation of different patients’ responses to drugs in a personalized manner.

A measure of ADPKD response to drugs can be formulated in terms of cysts number and their dimension. However, the lack of an automated system to quantify cysts in engineered kidney tubules did not currently allow the platform to be further developed and used at a large scale. As a way to estimate these quantities, we introduce the main contribution of this study, *i.e.*, the development of an artificial intelligence (AI) system to perform a fully-automatic segmentation of cysts on engineered kidney tubules to improve and automatize the detection and quantification of cyst number and size.

The development of automatic tools allowing for the precise detection and quantification of the structures of interest in medical images, going beyond a mere visual assessment, is of paramount importance in all medical imaging applications. Furthermore, this is particularly true when the data is made of many images containing many such structures that need to be measured. In recent years, several studies have explored the application of AI and Deep Learning (DL) techniques for the detection and characterization of diseases in physiological images^9^. A significant breakthrough in this area was the introduction of the UNet architecture by Ronneberger and coauthors^10^, a popular DL-based method for biomedical image segmentation. UNet has been widely adopted and adapted for various biomedical applications, including glands, lungs, and nuclei segmentation^11–13^. Based on this novel structure, many variations have been developed for medical image segmentation. Among the most widely used modified versions is the UNet++ architecture, which incorporates nested and skip pathways to enhance the model’s feature extraction capabilities^14^. This architecture has been applied to the segmentation of abdominal computed tomography (CT) scans and polyps, yielding improved performance over the original UNet. Another notable architecture is the UACANet^15^, which employs a self-attention mechanism to better capture the long-range dependencies within the input image, enabling the model to focus on the relevant regions for cyst segmentation. This self-attention mechanism also introduces a certain degree of explainability, which can be advantageous for the further development of the algorithm. The UACANet has demonstrated superior performance in polyp segmentation compared to other state-of-the-art architectures^15^. Focusing on imaging data of renal diseases, a long track record of publications^16^ can be found in the literature for segmentation^17^ and other tasks^18^, but only in the last few years, the polycystic kidney emerged as a topic of interest^19^, even in the private sector beyond academia^20^. The segmentation of specific tissues in an image is a common problem in medical image analysis, and many algorithms have been developed and made available^21–24^. The most addressed image analysis task in ADPKD, justified by the potential clinical applications, is the segmentation of cysts and kidneys at the macro-scale in magnetic resonance (MR) and CT images. The aim is to calculate indexes like kidney volume^25^ and cysts ratio^26^, helping to identify the stage of the disease. This task has been addressed with various semi-automatic^27^ and automatic^28,29^ methods. Little efforts have also been made on problems at the mesoscale by segmenting cysts on histological samples^30^. To the best of our knowledge, no studies have been conducted at the micro-scale on fluorescent images of ADPKD. Nevertheless, the research in drug discovery would greatly benefit from developing high-throughput screening platforms based on the analysis of images at the cellular level^31^. Various solutions exploiting machine learning for cell image analysis have been proposed^32^, also for fluorescent image analysis^33^, mostly adopting standard image processing techniques in a semi-automatic framework. However, in the last decade, deep learning became the state-of-the-art solution in many domains^34^, including kidney care^35^ and more in detail, cellular imaging with both optical^36^ and confocal microscopes^37^. In this work we investigate whether the segmentation of cysts in microscope images of patient-derived polycystic tubules can be addressed with the most recent DL-based solutions through a quantitative comparison of the state-of-the-art architectures targeting such task. The final aim is the design of a high-throughput screening platform supporting the researcher with the automatic annotation of images, and this study represents an effective first step toward this goal.

## Dataset and material

The dataset provided by *Istituto di ricerche farmacologiche Mario Negri* (Bergamo, Italy) is made of RGB immunofluorescence images of tridimensional human tubules engineered from epithelial cyst-lining cells that were isolated from a single donor patient with a mutation in PKD1. Tubules are the result of 4 individual experiments conducted from July 2019 to December 2020 and are classified according to the treatment received.

### Tubule engineering and cyst formation

Tubules were engineered by seeding huADPKD that were isolated from patient’s cysts on 3D printed PDMS scaffolds as previously described^7^. Briefly, huADPKD (purchased from Discovery BioMed Inc., Birmingham, AL, USA) were expanded on permeable, clear polyester filter supports ($Cat\#3450$, Corning) in DBM RenalCyte Specialty Medium (Discovery BioMed Inc.). When cells were confluent, they were harvested using $0.05\%$ (1x) Trypsin-EDTA ($Cat\#15400054$, Invitrogen), resuspended in 2.4*mg*/*ml* rat tail collagen type I ($Cat\#354236$, Corning, Corning-Costar, NY, USA) on ice at a concentration of $2\cdot 10^5 \,cells/uL$ collagen, and seeded in Polydimethylsiloxane (PDMS) scaffold’s channels (Sylgard 184 Silicone elasto-mer kit, Dow Corning, Midland, MI). Tubules were cultured under static conditions for up to 2 days in a standard incubator at $37\;^o$C with $5\%$ $\hbox {CO}_2$ and $20\%$ $\hbox {O}_2$ in DMEM/F12 + GlutaMAX supplemented with $1\%$ Fetal Bovin Serum (FBS) ($Cat\#10270$, Invitrogen Corporation, Carlsbad, CA, USA) and 40*ng*/*ml* hepatocyte growth factor (HGF) ($Cat\#100-39$, PeproTech), and then transferred onto polyester Transwell membranes (Corning) containing 200*uL* of 2.4*mg*/*ml* rat tail collagen type I. Cyst formation were induced by adding $10\, \mu M$ forskolin ($Cat\#F6886$, Sigma-Aldrich) to the culture medium (DMEM/F12 + GlutaMAX supplemented with $1\%$ FBS) for 2 days. Finally, tubules were treated for the following three days with specific compounds to revert cystogenesis. Control samples were stimulated with forskolin for 2 days and then maintained in culture medium for additional 3 days. For more technical details and visual representation of the protocol read Refs.^7,8^.

### Immunofluorescence and image acquisition

Engineered tubules were fixed with $4\%$ paraformaldehyde (PFA) ($Cat\#157-8$, Electron Microscopy Sciences) and permeabilized in $100\%$ cold methanol for 10 minutes. After washing, tubules were incubated with mouse anti-E-cadherin ($Cat\#610182$, BD Biosciences, 1:50) overnight at $4^{\circ }\text {C}$ and then with the specific secondary antibody (Jackson ImmunoResearch Labs, 1:50) overnight at $4^{\circ }\text {C}$. DAPI (Sigma-Aldrich) was applied for 10 minutes to staining the nuclei of the cells, and then the samples were mounted with Dako Fluorescence Mounting Medium (DAKO Corporation). Digital z-stack images of the whole tubular surface were acquired for both sides of the tubules using an inverted confocal laser microscope (Leica Biosystems). For each acquired image, cysts were manually annotated by using Labelme^38^ through a polygonal segmentation. Images composing the dataset and related information are intellectual properties of Istituto Mario Negri.

### Data characterization

The dataset consists of 1076 images of microscope acquisitions with a fixed scale and fixed size of $1024\times 1024$ pixels. The total number of annotated cysts is 5042. Table 1 shows the cardinality separated by period.

**Table 1:** Summary of the experiments.

Experiment	Period	# Treatments	# Tubules	# Images	# Cysts
1	September 2019	6	9	117	490
2	October 2019	7	7	103	216
3	July–September 2020	5	8	349	1589
4	December 2020	4	8	507	2767
Total	2019–2020	11	32	1076	5062

Some treatments are duplicated across experiments, the total value counts the unique treatments.

**Figure 1:**
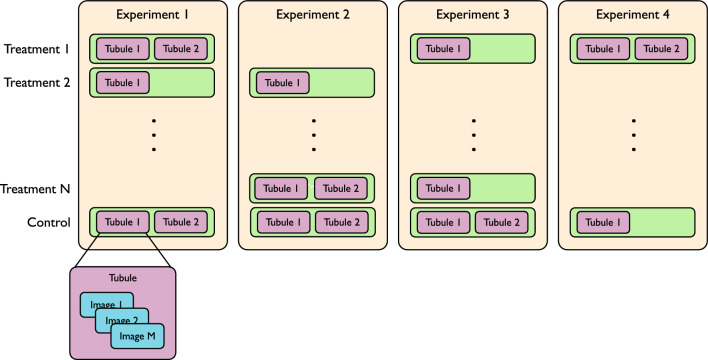
Experimental layout across different treatments. The treatments vary across experiments, but a treatment may be repeated multiple times. For each treatment or control, a maximum of 2 tubules are gathered. Each tubule is exclusively linked to a single experiment. Therefore, for example, tubule 1 in experiment 1, treatment 1, is distinct from tubule 1 in experiment 1, treatment 2, as well as tubule 1 in experiment 3, treatment 1. Multiple images are collected for each tubule.

Each of the four experiments comprises the study of various treatments (not always the same ones) and a control group. For each treatment or control, up to two tubules have been produced. For each tubule, various images have been acquired at different depths and viewpoints over the tubule. Each experiment used a different distance between the z-stacks of the acquisitions. Figure 1 shows the relationship between experiments, treatments, tubules, and images.

#### Cyst-scale problem

**Figure 2:**
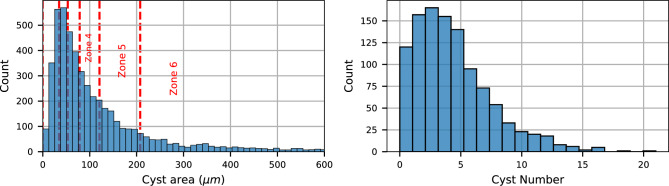
Dataset statistics. Statistics for cyst size (left side) and number of cysts per image (right side) on the whole dataset.

The cyst dimensions span a broad range of values over different images. The upper part of Fig. 2 shows the distribution over the whole dataset, depicting that smaller cysts located around $30~\mu m^2$, while the biggest ones reach $1900~\mu m^2$. The median of the distribution is around $78~\mu m^2$, which means it is highly peaked near the lower part of the graph. To address this peculiarity, we defined six adjacent zones with the same cardinality, i.e., approximately 840 cysts each, following the cyst area distribution: cysts with a size smaller than $34.7~\mu m^2$ fall into zone 1, the other zones start at $34.7~\mu m^2$, $53.3~\mu m^2$, $78.7~\mu m^2$, $120.9~\mu m^2$ and $207.5~\mu m^2$, respectively. The last zone includes cysts with a size up to $2000~\mu m^2$. The zones of the smallest cysts are around 20-30 $\mu m^2$ large, whereas the zone range significantly increases for bigger cysts. A similar behavior is also reflected in the number of cysts per image, reported on the right side of Fig. 2. Generally, the number of cysts in an image is close to the distribution median of four. However, there are some images with a very high number (e.g., more than 10) and others with no cysts at all. In the following sections, we will mitigate this imbalance by aggregating evaluation metrics over all images within a tubule.

**Figure 3:**
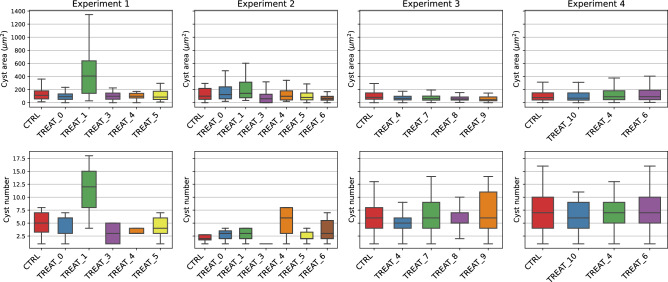
Statistics for treatment and experiment. Columns of the plot collect the data over the different experiments, each bar is an administered treatment. In the upper row we evaluate the cyst area, in the lower row the number of cysts is reported.

A closer look at these features shows that such a large variance is to be attributed to the difference between treatments. Figure 3 collects the previously discussed statistics separated by experiment and treatment. We can observe from the top row that a significant component of the large-sized cysts comes from TREAT_2 in experiment 1. Such an experiment has a diverging distribution also concerning the number of cysts per image. Such a large variance in the dataset is potentially challenging for the deep learning models. Hence the model evaluation will have to address this issue.

## Method

Deep learning algorithms can provide powerful and effective solutions for automated segmentation of polycystic kidney tubules. Different architectures have indeed been proposed in the literature in the last few years. Here we comparatively review some of the state-of-the-art segmentation approaches, using as a testbed the suite of RGB sequential immunofluorescence images from 4 in vitro experiments consisting of 32 engineered polycystic kidney tubules.

**Figure 4:**
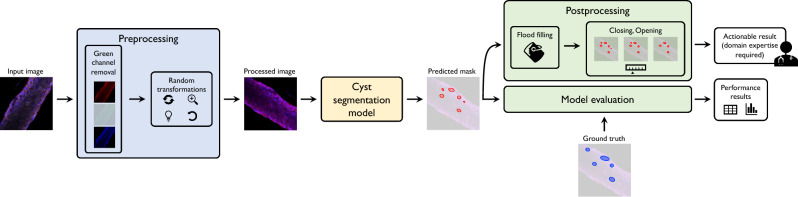
Experimental pipeline. Data flow of the application of Artificial Intelligence techniques for automated segmentation of engineered polycystic kidney tubules.

The experimental pipeline is presented in Fig. 4. It consists of three main steps: (i) an initial pre-processing phase that is aimed at data cleaning and augmentation, (ii) the application of a cyst segmentation model aimed at identifying the cysts, and (iii) a post-processing phase to support the analysis by a domain expert for the final evaluation. Each of these steps is described in further detail in the following subsections.

### Preprocessing

The preprocessing steps are applied to the input images to prepare them for the training of the cyst segmentation model. This data preparation is aimed at removing noise that is potentially contained in the images and augmenting them: we want the final model to be invariant to some operations, such as image rotations, orientations, and brightness.

#### Green channel removal

**Figure 5:**
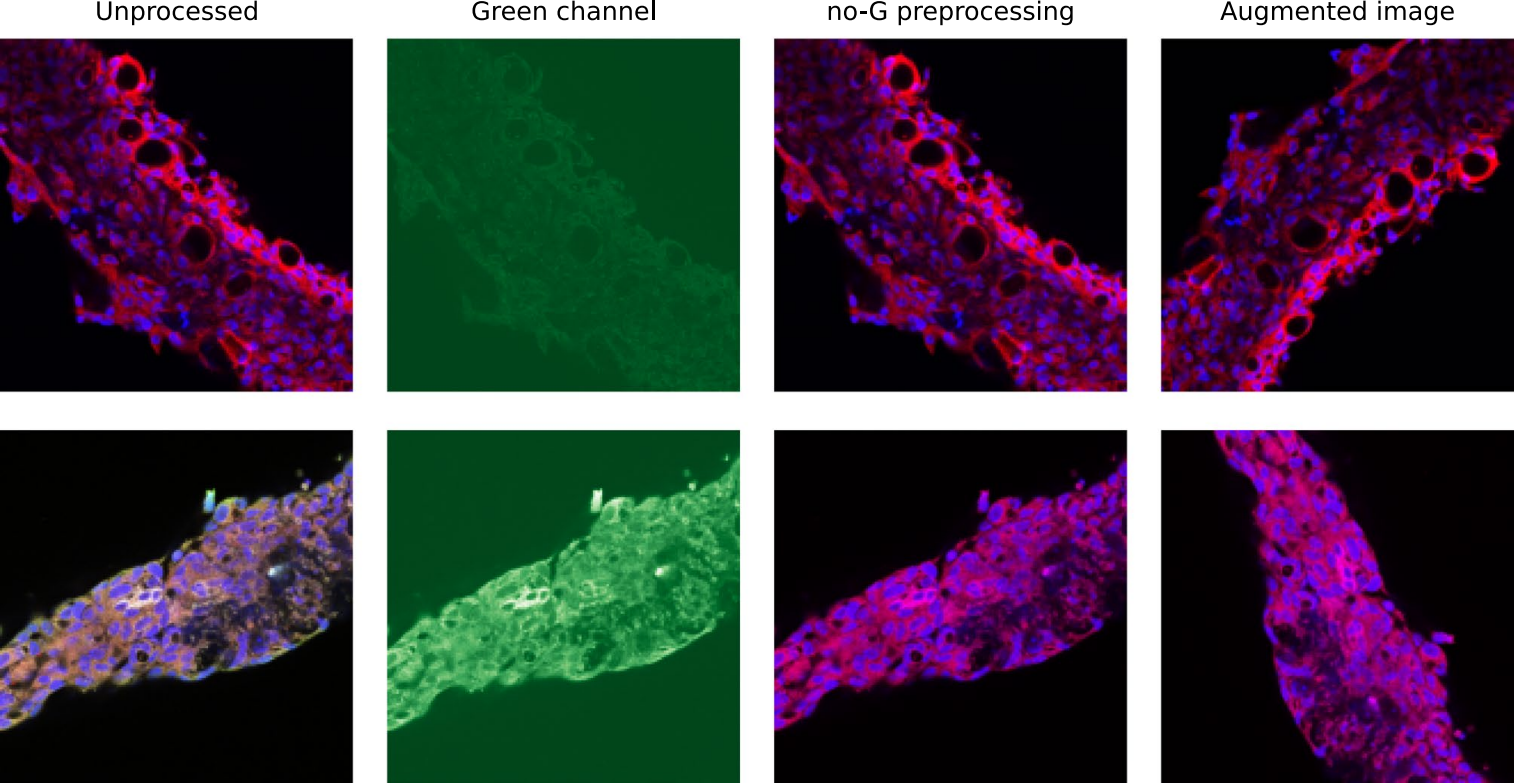
Tubule acquisitions and preprocessing. Rows represent 2 sample tubule acquisitions. The first column shows the raw images, and the second and the third are the associated green channel and the output image after muting the green channel. The last column shows the final image that is provided as input to the segmentation model after applying additional image augmentation techniques.

The images composing the dataset are generated after the application of highlighting fluorophores to the tissues to emphasize the most relevant components concerning the background. Human annotators generally recognize cysts as void globular holes surrounded by nuclei in the cell tissue. In order to help in the identification task, a red fluorophore is applied to spotlight the tissues and, as a consequence, the overall shape of the tubule. A blue fluorophore is then applied to stain the nuclei. Other markers are applied to some tubules to emphasize different points of the acquisition, but they are not relevant to the proposed analysis. Because of the colors of the fluorophores applied, we make the assumption that the most relevant information is found in the red and blue channels of the RBG images (Red Green Blue), with the green channel mostly containing noise or information that is unnecessary for the segmentation. The first preprocessing step applied is thus the removal of the green channel from the input images. The removal of the green channel can be accomplished in two ways: either by preserving the green channel and setting all of its values to 0 or by removing the channel altogether (i.e. producing RB images). Although the two approaches conceptually reach the same result, we decided to preserve the “muted” green channel for compatibility with later segmentation models that have been pretrained on (and thus expect as input) 3-channel images. Figure 5 shows two examples of unprocessed images, green channel, and output images after removing the green channel. We refer to this strategy as *no-G preprocessing* in the following.

#### Random transformations

Since deep learning solutions should learn the relevant features of the images neglecting non-fundamental ones, such as the direction of the tubules and the acquisition luminance, we applied data augmentation techniques to improve generalizability. This is a commonly adopted technique in computer vision to increase the number of available samples and train models to acquire some desired invariance properties^39^. Images used for training are thus subject to random transformations with different probability rates – as summarized in Table 2. A final augmentation step is applied to all images to normalize them so that they end up having the same RGB distribution as images from ImageNet^40^. Although this normalization does not affect the overall performance obtained, we achieved faster model convergence in this way. This happens because, after normalization, the inputs follow the same distribution as the data (ImageNet) already used for the pre-training the models. The last column of Fig. 5 shows two examples of augmented images.

**Table 2:** Random data augmentation techniques applied to dataset images to improve generalizability during the deep-learning model training.

Augmentation	Parameters	Probability
Horizontal flip	–	0.5
Random rotate	$\pm 90^\circ$	0.5
Random brightness	Brightness limit: 0.2	1.0
Random contrast	Contrast limit: 0.2	1.0
Random gamma correction	Gamma limit: (80, 120)	0.5
Contrast limited adaptive histogram equalization (CLAHE)	Contrast limit: 4.0	0.5
Normalize	ImageNet mean and stdev for each RGB channel	1.0

### Cyst segmentation model

The augmented images are used to train a deep-learning model that performs a segmentation task. The target for this segmentation task is a binary mask that labels each pixel as either belonging to a cyst or not. The ground truth was obtained through manual labeling. We compared 5 state-of-the-art image segmentation models to identify the best-performing deep-learning model, as described in the following.

- *UNet*^10^, a widely used model in medical image segmentation thanks to its effectiveness in combining low- and high-level features, and thus balancing the trade-off between architecture complexity and segmentation performance^41,42^. This model consists of two *macro-blocks*, the encoder and the decoder, connected through a series of extra convolutional blocks which act as *skip connections* between the encoder and the decoder. As in many other similar architectures, U-Net uses an initial contracting path (used to capture meaningful information) followed by an expanding one (to build an output with a similar shape as the input). Skip connections are used as stabilizers, reducing the loss of information that may incur in the encoding-decoding procedure and providing bypasses for the backpropagation of gradients.
- *UNet++*^14^, an improvement of the original U-Net model. It exploits the skip-connection strategy benefits by adding extra paths connecting the encoder and the decoder, thus further reducing the gap between the two blocks. Both UNet and UNet++ architectures are implemented with a ResNet50 encoder, pretrained on ImageNet^40^.
- *HardNet-MSEG*^43^, a HarDNet-based^44^ segmentation model, which uses Receptive Field Blocks for the decoding phase. HardNet-MSEG has been used for the task of medical image segmentation in the identification of colorectal adenomatous polyps.
- *PraNet*^45^ (Parallel Reverse Attention Network) is a model that follows a different paradigm than that used in previous networks. Input features are aggregated by a parallel partial decoder that generates a global map that is passed to a set of recurrent reverse attention modules. These modules are beneficial for extracting relationships between boundaries and areas. Similar to HardNet-MSEG, PraNet was also tested on a polyp segmentation task.
- *UACANet*^15^ is a model based on the PraNet architecture. It differs from PraNet mainly by using Uncertainty Augmented Context Attention (UACA) modules instead of Reverse Attention modules. UACA modules introduce a self-attention mechanism that incorporates uncertain regions to extract rich semantic features without introducing additional boundary guidance. UACANet has been shown to perform better than PraNet on a number of polyp segmentation tasks.

The output of each segmentation model is a 1-channel image with the same shape as the input, representing the predicted probability for each pixel to be a cyst. To obtain a binary mask, we discretized with a threshold of 0.5. This value is a good compromise between the accuracy of the model against unwanted cysts and the recognition of actual cysts.

**Figure 6:**
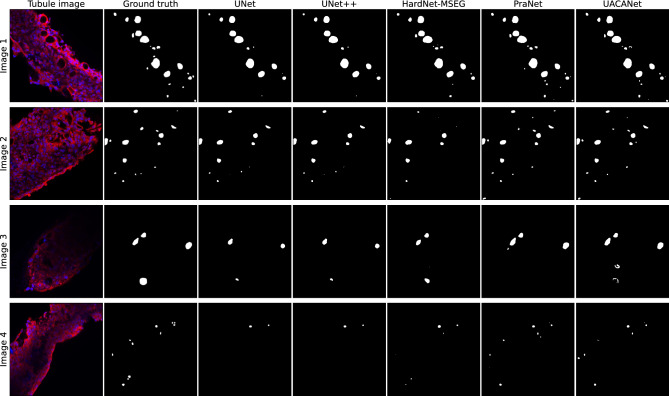
Segmentation results generated by the different models. The original tubule and ground truth mask are given for each of the 4 example images (one for each line row). The models’ predictions mostly agree with each other and the ground truth, especially for larger cysts. Smaller cysts are sometimes not detected or incorrectly predicted even though none are present.

Figure 6 showcases some examples of segmentations performed by the various models adopted. For each image, the original tubule and the manually annotated ground truths are reported, as well as the binary masks produced by each of the models. It can be observed how, despite minor behavioral differences, the models are primarily consistent with one another in their predictions. The experimental section will present quantitative results regarding the quality of the segmentation outputs.

### Training and tuning

Each model was trained using a binary cross- entropy function between the predicted mask and the expected binary output. We used an Adam optimizer and cosine annealing with warm restart to periodically decrease the learning rate and restart from its initial value across training epochs. This strategy was shown to improve the training speed^46^. Hyperparameters (e.g., learning rate) were set separately for each network configuration. Following a Bayesian search strategy, we found that the models with a learning rate of $10^{-4}$ and a stack size of 8 images achieved optimal performance within the available computational resources. An early stop strategy (with a maximum number of epochs of 100) was applied to stop training when no further improvement in Intersection-over-Union (IoU) was observed in the validation set. A summary of the main hyperparameters used for all models is shown in Table 3.

**Table 3:** Values of the main hyperparameters.

Hyperparameter	Value
Batch size (images)	8 images
Pretraining data	ImageNet
Optimizer	Adam
Learning rate	$10^{-4}$
Learning rate scheduler	Cosine Annealing with warm restart
Scheduler parameters	$T_0=10$, $T_{\textrm{mult}}=2$
Early-stopping patience	10 epochs
Maximum number of epochs	100 epochs
Binary threshold	0.5

All models have been fine-tuned using the same configuration.

### Model evaluation

Results for the best models are validated using a cross-validation strategy designed to both preserve the various levels of stratification of data and avoid data leakage. This second aspect stems from the observation that images from the same tubules are significantly correlated with one another. For this reason, we enforce placing all the same-tubule images in the same fold. The policy of separation was to keep together each image from the same experiment with the same treatment. Images belonging to the same experiment but different treatments (or vice versa) may be assigned to different folds: this is a necessary measure to guarantee same-size folds. This results in 32 folds, each containing images of one single tubule. We arrange them to form the *train-validation-test* split following a *LOTO* (Leave One Tubule Out) separation. This means that in each training pipeline, a model is trained and validated on 31 tubules (splitted in 80% training and 20% validation sets) and tested on the remaining one. In this process, the validation set is used to identify the early stopping point over the IoU curve for epochs, and final model weights are assigned based on this metric. Given the limited size of the dataset available, the LOTO approach (a specialization of the leave-one-out one) can be used with only limited computing power and provides the best possible estimate of the quality of the model on unseen data (i.e. how well the model can generalize to tubules other than the ones already seen).

We are interested in predicting both the number of cysts and their size accurately. Segmentation models are generally evaluated in terms of pixel-wise metrics, such as Intersection over Union (IoU), Recall (Re), and Precision (Pr). These metrics can be computed based on the ground truth value of each pixel (i.e. whether it is a cyst or not) and the value predicted by the model for the pixel (i.e., whether the model predicted that that pixel was a cyst or not). Table 4 shows the definitions for pixel-wise IoU, precision, and recall. In this context, true positives (TP) are cyst pixels that have been correctly labeled as cyst pixels, false positives (FP) are non-cyst pixels that have been erroneously labeled as cysts and false negatives (FN) are cyst pixels that have not been detected as being cysts (i.e., they have been labeled as non-cysts).

Pixel-wise metrics provide useful information about the overall quality of the model. However, we note that these metrics tend to weight cysts with larger areas: Since these larger cysts consist of a larger number of pixels, their correct detection has a greater impact on the metrics than smaller cysts.

For this reason, we additionally propose the use of cyst-based metrics by extending the used pixel-wise metrics to a cyst-based granularity level. In this way, we can weight all cysts equally regardless of their area. In our previous work, we have also introduced and discussed these metrics^47^.

**Table 4:** Pixel- and cyst-wise evaluation metrics considered from the confusion matrix.

Metric	Pixel-wise	Cyst-wise
Intersection over Union (IoU)	$\frac{TP}{TP+FN+FP}$	$\frac{DT}{DT+MS+WR}$
Precision (Pr)	$\frac{TP}{TP+FP}$	$\frac{DT}{DT+WR}$
Recall (Re)	$\frac{TP}{TP+FN}$	$\frac{DT}{DT+MS}$

We first define a notion of *overlap*: a predicted cyst and a ground truth cyst are considered to be overlapping if they have some pixels in common (e.g., as a threshold value on their pixel-wise IoU). We will study the impact of this choice of threshold in the experimental section. Based on this notion, we can identify a ground truth cyst as being *detected* (DT) if it overlaps with at least one predicted cyst. If no predicted cyst overlaps with it, the ground truth cyst is instead referred to as *missed* (MS). If a predicted cyst does not overlap with any of the ground truth cysts, it is labeled as *wrong* (WR). These values are the counterparts of true positives, false negatives, and false positives respectively. We note that a cyst may be detected multiple times (i.e. it may overlap with multiple predicted cysts). To avoid inconsistencies, we consider this situation as one detected (DT) cyst and the rest as *wrong* (WR). Similarly, a predicted cyst that overlaps N ground truth ones is considered as one detected (DT) cyst and N-1 missed (MS) ones.

**Figure 7:**
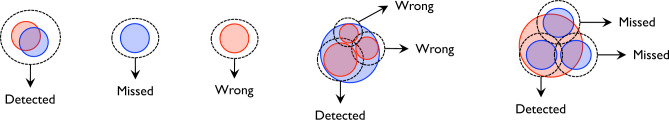
Cases of overlap between real (blue) and predicted (red) cysts. Examples of overlaps between ground truth cysts (in blue) and predicted cysts (in red). Each situation is identified with the type of label that is assigned to it.

**Figure 8:**
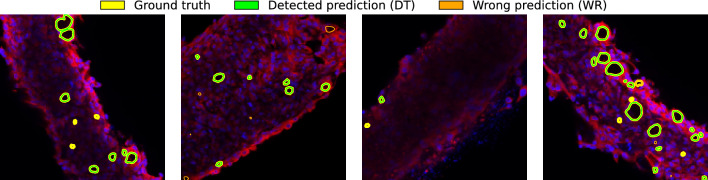
Predictions from four different input images. For each image, contours of manually annotated (DT and MS) and predicted cysts (DT, WR) are highlighted. Yellow contours identify the ground truth cysts, they are DT if there is a predicted (green) contour over them, MS otherwise. Incorrect predictions are marked as WR.

Figure 7 exemplifies the various types of situations that can occur between predicted and ground truth cysts. Figure 8 additionally illustrates some examples of cyst predictions and ground truths, with examples of detected, missed, and wrong cysts. Table 4 additionally contains the definitions of IoU, precision, and recall when computed at the cyst level.

### Postprocessing

After obtaining a binary mask from the segmentation model, we apply two postprocessing steps to make the segmentation output more useful for the domain expert who will assess and evaluate the result. The first postprocessing step consists in filling holes that may occur within some of the segmented images. We reasonably assume that cysts do not typically contain holes within them. Based on this, all pixels of the predicted mask containing “non-cyst” pixels surrounded by “cyst” pixels are automatically switched to “cyst” pixels. We accomplish this through a simple flood-fill algorithm.

The second (optional) postprocessing step consists in either applying an *opening* or a *closing* morphological operation, which act as follows:

- *Closing* consists in applying a dilation operation followed by an erosion one. The two operations are applied pixel-wise and consist in replacing each pixel value with either the maximum (dilation) or minimum (erosion) that are found within a neighborhood of the pixel itself. The closing operation results in an image where neighboring clusters of pixels (cysts) are merged together.
- *Opening* applies an erosion followed by a dilation. This order of operation makes small clusters of pixels disappear due to the application of the erosion operation. This operation can be useful to remove noisy segmentations (i.e., predicted cysts that are only a few pixels in size)

Both operations rely on the notion of a neighborhood. This neighborhood is defined through a structuring element, whose size and shape determine the specific properties of the openings and closings. More specifically, we let the size of the structuring element *k* as a parameter that the domain expert can control to decide the extent of the desired behavior. Instead, we define the shape of the structuring element as circular, given the assumption that we expect cysts to have a circular shape.

**Figure 9:**
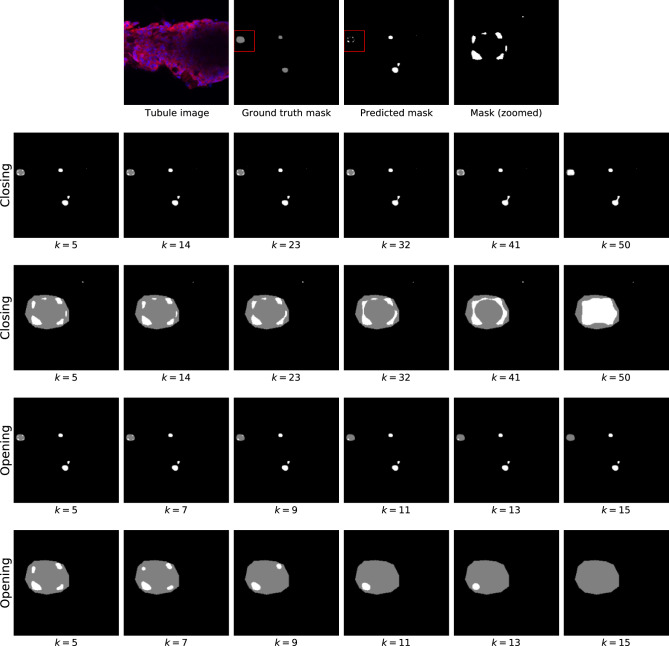
Example of postprocessing on a sample image. The first row includes the original image, the ground truth, and the mask predicted with UACANet. The second and third rows show the effect of applying the *closing* technique, whereas the fourth and fifth rows show the effect of applying *opening*. The results are shown both for the entire image (second and fourth rows) and for a close-up of an interesting case (third and fifth rows). A red square is used in the top row to identify the portion of the image used for the close-up. For all images, the ground truth is shown in grey, the prediction is shown in white.

Figure 9 shows the application of an opening or closing application on a sample image, using different *k* values. We observe how applying a closing can mitigate the overcounting problem, which occurs when the model segments one of the real cysts as multiple ones. Closing can be used to make the various predicted cysts merged into a single one.

The opening operation can be used instead to remove small (noisy) segmented cysts. For example, in the zoomed mask, a small cyst can be seen in the upper right corner of the image. While this cyst cannot be removed by closing it, it quickly disappears when opened with a small value for *k*. Using this example, we can also see some problems that can occur when opening and closing. Excessive closing can cause cysts that were previously correctly separated to be incorrectly merged (as with the lower cyst in the example image). Excessive closing, on the other hand, could result in small – but correctly identified – cysts no longer being recognized as such. Based on these considerations, we believe that both opening and closing cysts can be a helpful support if used carefully. In this paper, we leave the option of applying these steps (and, if so, the extent to which they are applied) to the expert analyzing the results. We additionally present some results of applying either approach in the experimental section. However, we are aware that this post-processing could be applied differently in different sections of the image (e.g. different operations, different *k* values): one of the future directions of this work will be to automate this post-processing step.

## Experimental results

In this section, we present the results of the LOTO cross-validation applied to each segmentation model. The main results show the performance of the various segmentation models without postprocessing. We additionally study the behavior of the best-performing model as a function of the size of the cysts. We show that the proposed method has a consistent behavior throughout treatments, which implies that the proposed methodology is robust to changes in the distribution of cysts’ number and size. Finally, we observe how applying the proposed opening and closing postprocessing techniques impacts the performance.

### Deep learning model comparison

Table 5 reports the model performance in terms of IoU, precision, and recall for both the pixel- and cysts-wise metrics. We can observe that all the models can reach comparable results in terms of pixel- and cyst-wise metrics. UACANet is the model that generally performs best regarding IoU and recall. In terms of precision, we instead observe the best results with UNet++. Looking at the cyst-wise measures, the interpretation is that UACANet tends to over-estimate the presence of cysts (thus achieving higher recall and lower precision), whereas UNet++ makes more conservative estimates, only predicting cysts when there is high confidence (resulting in lower recall, but higher precision).

Most confidence intervals of the results overlap with one other. Concerning IoU-like metrics, UNet, UNet++, and PraNet get values close to UACANet. For the precisions, all models except PraNet approach the best-performing one, i.e., UNet++. PraNet is close to the best model in the recall metrics. Even though all the models achieved comparable results, UACANet is found to be, on average, the best-performing architecture overall. For this reason, in the following, we elect UACANet to be our reference model.

**Table 5:** Performance of the cyst segmentation models in terms of Intersection over Union, Precision, and Recall, both pixel- and cysts-wise.

Model	IoU	Pr	Re	$\hbox {IoU}_{\textrm{cyst}}$	$\hbox {Pr}_{\textrm{cyst}}$	$\hbox {Re}_{\textrm{cyst}}$
UNet	$0.5845\pm 0.0451$	$0.8484\pm 0.0305$	$0.6607\pm 0.0522$	$0.6029\pm 0.035$	$0.7743\pm 0.0448$	$0.7463\pm 0.0353$
UNet++	$0.5821\pm 0.0525$	$\mathbf {0.8591\pm 0.0288}$	$0.6492\pm 0.0577$	$0.6059\pm 0.0493$	$\mathbf {0.8385\pm 0.0321}$	$0.696\pm 0.0554$
HardNet-MSEG	$0.5311\pm 0.0527$	$0.8564\pm 0.0221$	$0.5893\pm 0.06$	$0.5664\pm 0.0453$	$0.787\pm 0.0324$	$0.6812\pm 0.0516$
PraNet	$0.5868\pm 0.0493$	$0.8108\pm 0.0311$	$0.6896\pm 0.0574$	$0.6224\pm 0.0369$	$0.7171\pm 0.0372$	$0.8276\pm 0.0323$
UACANet	${\textbf {0}}.{\textbf {6239}}\pm {\textbf {0}}.{\textbf {0359}}$	$0.8339\pm 0.0333$	**0**.**7128**±**0**.**0352**	**0**.**6464**±**0**.**0395**	$0.7406\pm 0.0411$	**0**.**8346**±**0**.**0262**

Best values for each metric are in bold.

### Overlaps between predictions and ground truth

When defining the cyst-wise metrics, we made the assumption that a cyst is detected (DT) if the overlap between a predicted cyst and a real one (as quantified by the IoU between them) is above some threshold value.

**Figure 10:**
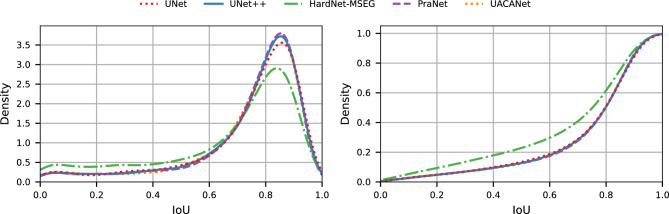
IoU between detected (DT) cysts and their real counterpart. The left figure shows the distribution, while the right one is the cumulative of the same quantity.

To identify a meaningful IoU threshold for the definition of detected cases (and, as a consequence, the other metrics), we study the distribution of IoU values between predicted and ground truth cysts. Figure 10 shows the distribution (as modeled with a kernel density estimation) of values for all detected cysts (assuming a minimum overlap of 1 pixel). We observe that most cysts are detected with a large overlap (95% of cysts are detected with an IoU greater than 0.2, and 80% of the cysts are detected with an IoU greater than 0.6). This implies that, if the model detects the presence of a cyst, its segmentation of the cyst itself will be particularly accurate. We additionally note that 65% of the cysts predicted with an IoU smaller than 0.2 are the smallest ones (zone sizes 1 to 3). As we will discuss, these cysts are the most problematic to detect. Larger cysts (i.e., those that are more relevant in terms of cyst size) are generally well segmented (i.e., they have a large IoU). Because of this, and with the overall goal of achieving good performance in terms of recall, we decide to consider a cyst detected if the predicted and ground truth cysts overlap at least 1 pixel.

### Cyst size

**Figure 11:**
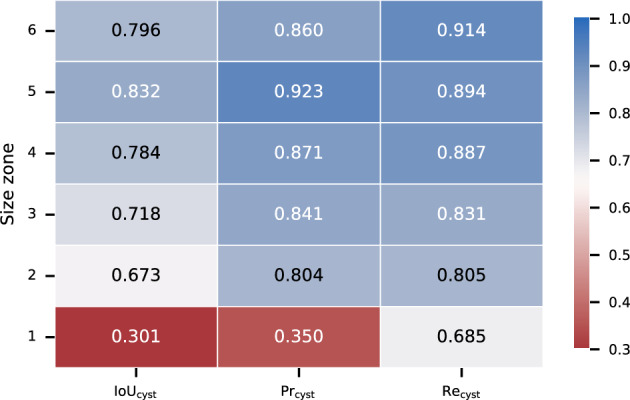
Cyst-wise performance by size. Performance is reported on the test set for UACANet, aggregated by cyst size.

Given the heterogeneous size range of the labeled cysts, we aggregated the results of the models by cyst size to determine if this affected the network’s learning ability. Focusing on the UACANet architecture, we evaluated the cyst-wise performance for the cysts in each of the 6 size zones (as described in Fig. 2) and presented the results in Fig. 11. For partitioning, the actual size is considered for DT and MS cysts, whereas the predicted size is considered for WR cysts (no information on the actual size is available because the predicted cyst does not exist). This means that a predicted cyst is considered for evaluation within the size zone of its ground-truth counterpart, if available, within its size zone otherwise.

We observe an increase in predictive power with larger cyst sizes. Precision and recall scores achieve similar performance throughout zones, except for the first zone, which contains the smallest cysts. In this case, the precision is significantly lower compared to the results for the other size categories. The recall for the smallest zone is still lower than for the other zones, but less significant. This can be explained by the fact that the model makes most false-positive (WR) predictions for small cysts. In other words, the cysts that the model incorrectly predicts are usually very small (mostly zone 1). We also note that some of the incorrectly-predicted zone-1 cysts were later accepted as valid cysts when reevaluated by human annotators, confirming that detection of small cysts is a difficult task even for humans.

### Treatment invariance

**Figure 12:**
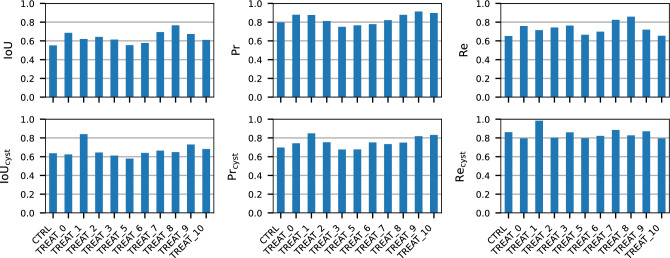
Performance measures separated by treatment. The upper and lower rows show the pixel- and cyst-wise metrics, respectively.

This work aims to investigate the feasibility of an affordable cyst detection platform. Therefore, we need a model capable of producing excellent and stable results regardless of tubule treatment. In this way, the effect of prediction error in evaluating treatments would be reduced if it is the same for all treatments. This property was evaluated by separating the results by treatments and comparing them using the proposed metrics in Fig. 12. We find that the highest difference in performance between treatments is at most 0.2 points for each metric. Furthermore, there is no meaningful correlation between these results and the statistics of the treatments presented in Fig. 3. In particular, the anomalous distribution of images from TREAT _2 does not affect the predictive capabilities of the network.

### Preprocessing impact

In Section , we presented the concept of *no-G preprocessing*, which incorporates expert-guided supplementary information indicating that the green channel’s content is irrelevant to the task. Demonstrating the advantages of integrating this knowledge for enhanced performance, Table 6 displays the outcomes of UACANet, identified as the top-performing model for this task, across all the experiments.

We note a general improvement in performance in all the experiments, but the most substantial improvement is seen in Experiment 3. In this particular experiment, it is worth noting that the experts involved in the acquisition of the tubules reported that the green channel shows additional fluorophores unrelated to the scope of this study. It should be reiterated that this technique is specifically tailored to this particular task. Nonetheless, these results highlight the advantages of using a custom pipeline over general segmentation algorithms that are readily available.

**Table 6:** Performance of the cyst segmentation models in terms of Intersection over Union, Precision, and Recall, both pixel- and cysts-wise.

	Exp. 1	Exp. 2	Exp. 3	Exp. 4
Without noG	0.6006 ± 0.0456	0.4757 ± 0.0667	0.4340 ± 0.0237	0.6960 ± 0.0153
With noG	**0.6087 ± 0.0446**	**0.4786 ± 0.0653**	**0.4814 ± 0.0246**	**0.7037 ± 0.0143**

Best results for each experiment are in bold.

### Postprocessing

**Figure 13:**
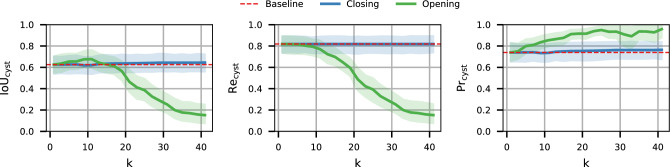
Effect of the opening and closing postprocessing at different values of $\varvec{k}$. The baseline result of the UACANet model without postprocessing is reported by the dashed red line.

The results presented so far were obtained without applying the opening or closing postprocessing effects. As discussed earlier, these effects can be used to find a better compromise between precision and recall. Although we leave the decision on whether to use these techniques to domain experts (as well as the extent to which they should be applied), we nevertheless report on the results that can be obtained by applying either technique with different kernel sizes (*k*). This is to provide a general overview of the effect that the opening and closing techniques can provide. In particular, Fig. 13 shows the effect on precision and recall when using different *k* values, for both opening and closing. We observe two very different behaviors for opening and closing. When using closing, the effect is very small and not statistically significant across confidence intervals. However, we observe a slight increase in both IoU and precision, with a negligible effect on recall.

This behavior occurs because some of the wrong (WR) cysts are the result of an ”overcount” in which a single ground truth cyst is segmented as multiple smaller cysts (one such case is shown in Fig. 9). As closing is applied, the smaller cysts are merged into fewer larger ones (third line in Fig. 9). As expected, this reduces the total number of false cysts found.

The opening postprocessing has instead the effect of removing the smallest cysts and separating the cysts that are close to each other and predicted to be together. We observe a steady increase in precision and a steady decrease in recall. Based on the previously stated consideration that many of the incorrectly predicted cysts are small, the observed increase in precision can be easily justified. The reduction in recall can be explained by the fact that the opening removes some of the small cysts that were correctly identified.

In summary, we can see that the effect of opening and closing can be beneficial in some cases, but detrimental in others. For this reason, we leave it to the expert to perform these operations as needed.

### nnUNet comparison

Our solution addresses the challenges the cyst segmentation task specifically poses. We extend our experimental comparison here, including a more generic alternative, the nnUNet^48^.

We conducted a thorough evaluation and found that our approach yields equal or superior results. Namely, our specific pipeline with the UACANet model reaches better performance in IoU ($+0.27\%$) and recall ($+2.77\%$), while nnUNet is superior in precision ($+6.48\%$). Notably, nnUNet requires a fixed training regimen of 1000 epochs, from which the best model is selected. This approach comes with substantial computational overhead, with average epoch times ranging from 50 seconds to a maximum of 200 seconds in some experiments, resulting in a minimum expected training time of at least 13 hours. In contrast, our pipeline with the UACANet model converges within just 30 epochs, with an average epoch time of 120 seconds. Saving almost 2 orders of magnitude in computational costs and reaching almost the same performance, we recognize the simplicity and flexibility of general models such as nnUNet, yet we consider that for the specific task, the proposed pipeline provides domain experts with a better overall experience thanks to the quick task completion (i.e., few minutes). LOTO cross-validation has been used to perform the comparison in an experimental setting as close to our framework as possible.

## Discussion and conclusion

This work presents an end-to-end experimental pipeline for cyst segmentation in immunofluorescence images obtained from engineered polycystic kidney tubules. We compare 5 state-of-the-art deep learning segmentation models (UNet, UNet++, HardNet-MSEG, PraNet, UACANet) on a testbed of 5062 cysts on 1076 images of 32 engineered polycystic kidney tubules from 4 *in vitro* experiments. The experimental design is based on a specific leave-one-out crossvalidation, named * LOTO * (Leave One Tubule Out): each model is trained and validated on 31 tubules and tested on the remaining one. Model performance was evaluated using Precision, Recall, and Intersection over Union, each specialized at both pixel and cyst-wise levels, and focusing on overlapping cyst detection.

The experimental pipeline includes common preprocessing steps such as data augmentation by random transformations and specific preprocessing steps such as green channel removal. Optional post-processing steps are also included in our pipeline and are experimentally evaluated. They aim to reduce noise in the segmentation results, e.g., by removing very small cysts or merging clusters of small cysts into larger cysts. The results show a trade-off between precision and recall. For this reason, we currently leave these post-processing steps as an optional feature that can be applied by experts if needed.

The core of the experiments aims at comparing the deep learning models. The UACANet model performed best, while all other models produced comparable results. The best pixel-wise performance for Intersection over Union is 0.624 of UACANet, which is 0.037 higher than that of the second best, PraNet; for Recall, UACANet achieved 0.713, which is 0.023 higher than the second best, PraNet; for Precision, UNet++ achieved 0.859, which is only 0.03 higher than the second best, HardNet-MSEG. The results show that the segmentation models perform poorly in detecting small cysts, which is consistent with the degradation in segmentation quality achieved by human annotators for similarly sized cysts, as expected. The best performing model, UACANet, achieves a cyst-wise Intersection over Union as high as 0.83, 0.91 for Recall, and 0.92 for Precision when applied to detect large-size cysts (average over a full * LOTO * cross validation ). Cyst-wise metrics are robust to occasional pixel overlap, as 80% of the predicted cysts have an Intersection over Union with respect to the ground truth greater than 0.6. The results are robust to treatment changes, meaning that the models are consistent when confronted with variations in the underlying distribution of cyst number and size.

The source code to reproduce all experiments is freely available on a public GitHub repository. The dataset is the property of Istituto Mario Negri and is available upon request.

As a future research direction, we aim to automate the post-processing steps with a self-assessment mechanism capable of selectively choosing different regions of the segmentation output for the application of appropriate parameters. We intend to extend the experiments to new polycystic kidney tubules to further assess the robustness and generalizability of the pipeline and the models themselves. Model extension is also part of our plans, in particular we are developing knowledge-based techniques capable of capturing cyst constraints and 3D mesh solutions for image segmentation.

## Data Availability

The code to reproduce all the experiments is available on a public GitHub repository at https://github.com/simone7monaco/auto-cysts-segmentation. Data underlying the results presented in this paper are property of Istituto Mario Negri, Milano, Italy; they are not publicly available at this time, but may be obtained from the corresponding author upon request.
