# Author Correction: AI models for automated segmentation of engineered polycystic kidney tubules

**DOI:** 10.1038/s41598-024-55389-8

**Published:** 2024-02-27

**Authors:** Simone Monaco, Nicole Bussola, Sara Buttò, Diego Sona, Flavio Giobergia, Giuseppe Jurman, Christodoulos Xinaris, Daniele Apiletti

**Affiliations:** 1 DAUIN, Politecnico di Torino, 10129 Turin, Italy; 2 Fondazione Bruno Kessler, 38123 Trento, Italy; 3 CIBIO, Università degli Studi di Trento, 38123 Trento, Italy; 4 Istituto di Ricerche Farmacologiche Mario Negri - IRCCS, 24126, Bergamo, Italy

Correction to: *Scientific Reports* 10.1038/s41598-024-52677-1, published online 03 February 2024

In the original version of this Article, Christodoulos Xinaris was omitted as a corresponding author. Correspondence and requests for materials should also be addressed to christodoulos.xinaris@marionegri.it.

The original Article has been corrected.

